# Development, characterization, and mechanical, dynamic mechanical, and hydrophilic studies of a sustainable *Aquilaria agallocha* gum-filled epoxy hybrid biocomposite reinforced with hemp and snake grass fibers

**DOI:** 10.1039/d6ra05721g

**Published:** 2026-08-26

**Authors:** N. Gopalakrishnan, B. Sridevi, R. Nalini Suja, M. Chitra, A. Subramani

**Affiliations:** a Department of Chemistry, Presidency College Chennai 600005 Tamil Nadu India drbsridevi73@gmail.com; b Department of Chemistry, Panimalar Engineering College Chennai 600123 Tamil Nadu India; c Department of Chemistry, Chellammal Women's College Chennai 600 032 Tamilnadu India; d Department of Chemistry, Dwaraka Doss Goverdhan Doss Vaishnav College (Autonomous) 833, Gokul Bagh, E.V.R. Periyar Road, Arumbakkam Chennai 600 106 Tamil Nadu India

## Abstract

Natural fiber-reinforced epoxy composites are increasingly popular as eco-friendly alternatives to synthetic materials due to their low density, biodegradability, and renewability. In this investigation, a unique hybrid epoxy biocomposite was produced using *Aquilaria agallocha* gum as a biofiller and snake grass and hemp fibers as reinforcement. Hand lay-up followed by compression molding was used to create composites with 15 vol% biofiller and varying fiber contents (5–30 vol%). A systematic study was conducted to determine how adding hybrid fiber affects mechanical, dynamic mechanical, water absorption, and biodegradation properties. At 20 vol% fiber loading, tensile strength peaked at 29.2 MPa, while impact strength peaked at 25 vol% with 2.7 kJ m^−2^. At 30 vol% fiber concentration, the highest flexural strength was 41.72 MPa. Dynamic mechanical analysis showed an increase in glass transition temperature to ∼80 °C and a rise in storage modulus up to ∼6.5 × 10^9^ Pa, suggesting improved fiber–matrix interactions and restricted polymer chain mobility. The inclusion of hydrophilic natural fibers was shown to result in controlled moisture absorption and partial biodegradation, as demonstrated by soil burial and water absorption tests. Effective fiber dispersion, interfacial adhesion, and synergistic hybridization effects are responsible for the improved characteristics. The produced composite shows promise for use in lightweight, sustainable structural applications that require a low carbon footprint, moderate strength, and ecological compatibility.

## Abstract 2

A sustainable epoxy-based hybrid biocomposite was successfully developed using *Aquilaria agallocha* gum as a biofiller and hybrid reinforcement of hemp and snake grass fibers.

## Graphical abstract

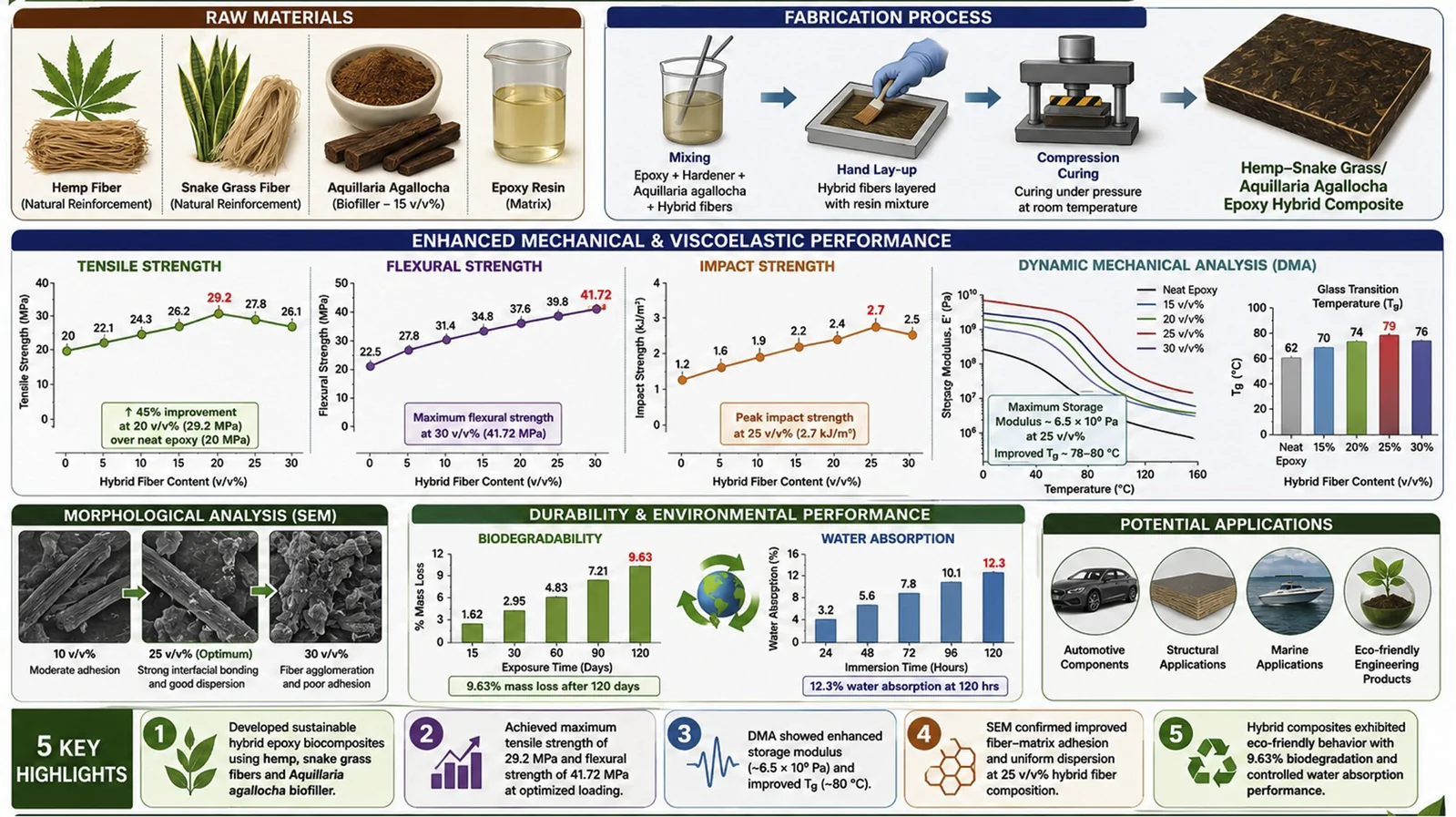

## Introduction

The rapid growth of the polymer industry has significantly increased the use of synthetic plastics and thermosetting resins, especially epoxy resins, which are highly valued for their exceptional mechanical strength, strong adhesion, low shrinkage, chemical resistance, and superior dimensional stability. These properties make them essential in aerospace, automotive, marine, electrical insulation, coatings, and structural applications. However, conventional epoxy resins are primarily derived from petroleum-based resources such as bisphenol-A and epichlorohydrin, raising serious environmental and health concerns. Their non-biodegradability, high carbon footprint, and limited recyclability have intensified efforts to develop sustainable alternatives, especially as many regions have limited single-use plastics.^1–5^

Natural fiber composites made from lignocellulosic resources offer benefits such as reduced energy use, lower greenhouse gas emissions, and environmentally sound disposal options, including recycling or biodegradation. They are attractive to industries such as automotive, construction, and packaging, aligning with circular economy principles. Rising demand for sustainable products drives researchers and automakers to focus on the ecological impact of materials during recycling and disposal, increasing interest in sustainable, biodegradable composites.^6^

Plant fibers are important for research and industry because of their affordability and robust properties. They contain cellulose, hemicellulose, and lignin. Cellulose provides strength, while hemicellulose enhances flexibility. Lignin adds rigidity and resistance to decay. Different types of fibers, such as cotton, jute, and banana, are used in textiles and composites for their specific strengths. Natural fibers are increasingly popular as alternatives to harmful materials in sectors like automotive and aerospace, offering improved performance. They also have applications in construction, geotextiles, and bioplastics. Despite their potential in biomedicine, challenges remain.^7–17^

Composites that are completely green, or bio-composites, are made from bio-fibers and resins from renewable sources to reduce environmental harm. There are two types: fully green composites with a bio-based matrix and partially green composites with a petrochemical matrix, both using bio-fibers. These composites are widely used in the military, automotive, aviation, and medical industries. Common natural fibers include jute, sisal, bamboo, coir, and snake grass, chosen for their lightweight and strength, despite drawbacks such as moisture absorption. Snake grass fibers have specific mechanical properties that improve with fiber length and volume. Common matrices include natural rubber, polyethylene, and epoxy, which help create eco-friendly composites. Hemp fiber, derived from hemp plants, has varying properties based on growth conditions. The plant genus *Aquilaria* includes trees that produce valuable resin, used in traditional medicine and showing potential health benefits.^18–21^ Hybridization of biocomposites is proposed to enhance the materials' strength and resistance, using combinations of different natural fibers. The research aims to explore the interaction between hydrophilic natural fibers and hydrophobic epoxy resin to assess adhesion, mechanical performance, and water resistance. Natural fiber-reinforced epoxy composites were successfully produced, and their properties were evaluated. The study emphasizes the integration of agricultural waste materials into eco-friendly epoxy composites, examining their mechanical and water-resistance performance, and showcasing their applications in construction and industry through Mechanical Testing, DMA analysis, and hydrophilic studies.

The study seeks to evaluate water resistance, mechanical performance, and adhesion by examining the interaction of hydrophobic epoxy resin with hydrophilic natural fibers. The properties of natural fiber-reinforced epoxy composites were evaluated, and they were successfully manufactured. The study focuses on incorporating agricultural trash into environmentally friendly epoxy composites, evaluating their mechanical and water resistance performance, and demonstrating their uses in construction and industry using mechanical, DMA, and hydrophilic investigations.

This study is the first thorough investigation of epoxy composites that have been strengthened concurrently with hemp fiber, snake grass fiber, and *Aquilaria agallocha* gum biofiller. In contrast to systems previously reported that primarily use single natural fibers or only fiber reinforcement techniques, the present work uses a bio-based particulate filler and dual-fiber hybridization to improve multifunctional performance. The research directly links microstructure, interfacial interactions, mechanical properties, dynamic mechanical behavior, moisture diffusion, and biodegradation characteristics. It also provides a practical design framework for sustainable structural bio composites by identifying an optimal fiber-loading window that balances toughness, stiffness, strength, thermal stability, and environmental compatibility.

## Experimental section

A polyamine-type hardener (HY951) and an epoxy resin (LY556) matrix material were used in this study. *Aquilaria Agallocha*, purchased from local vendors in Kanchipuram, India, served as the biofiller for this research. Snake grass fibres and Hemp fibres were procured from Go Green Products Valasaravakkam Chennai.

### Preparation of *Aquilaria agallocha* gum powder

*Aquilaria agallocha* gum powder procured from the vendor was dried, the powder was ground using ball milling method. The planetary ball mill was fitted with stainless steel jars and 10 mm stainless steel balls. Milling was carried out at 250 rpm for 5 h. To avoid excessive heating the experiment was conducted for 20 minutes milling and 10 minutes rest cycles till the total milling time of 5 h was achieved. The powder was then sieved which produced grains ranging from 10 to 25 microns in size. The biofiller made from powdered *Aquilaria Agallocha* has density of 1.051 g cm^−3^.

### Pre treatment of snake grass and hemp biofibres

Snake Grass and Hemp were washed with double distulled water to remove dirt and surface impurities. It is then followed by cold ethanol wash to remove waxes. The fibers dried in hot air oven at 60 °C for 24 h. The process of cleaning and drying was repeated until constant weight was obtained. The dried fibres are cut into 10 mm length and stored in sealed polyethylene bags and kept in dessicator until further Use.

### Development of biocomposite

The hardener and epoxy resin were mixed in a 10 : 1 weight ratio. Snake grass and hemp fiber were added to the epoxy mixture at predetermined volume fractions. To ensure even distribution of the fibres in the resin, the mixture was manually stirred for 15 minutes. This was followed by gently pouring the liquid into a wooden mold and carefully smoothing the surface with a brush. The cured composite was then cut into samples using ASTM dimensions to ensure consistency among the test specimens.

Curing reaction
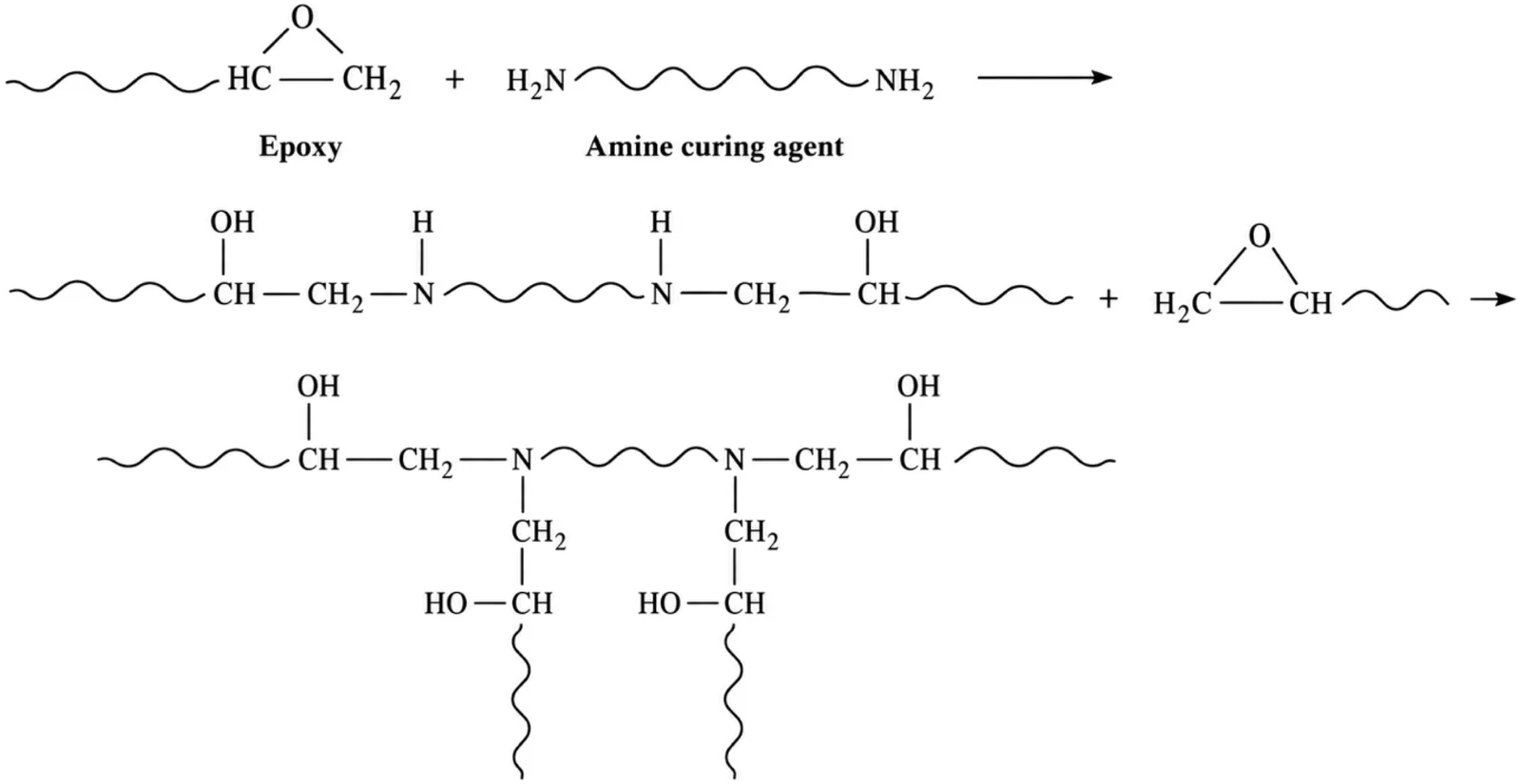


### Fabrication of *Aquilaria agallocha* gum with snake grass and hemp fibre reinforced epoxy composite

The compression molding method was used to create hybrid natural fiber reinforced composites that included *Aquilaria agallocha* gum bio-filler, hemp fibers, and snake grass fibers. Using a mould with dimensions of 250 mm × 250 mm × 3 mm thickness, the composite laminates were manufactured.

A ball milling process was used to transform *Aquilaria agallocha* gum into a fine powder. Ball milling was carried out at a rotational speed of 1200 rpm for two cycles to achieve a consistent particle size distribution. Scanning Electron Microscopy (SEM) analysis confirmed that the average size of the bio-filler particles was between 15 and 20 µm.

The HY 951 hardener and LY 556 epoxy resin formed the matrix system. Before fabricating the composite, the resin and hardener were mechanically mixed to produce a uniform hybrid resin mixture.

The mold was thoroughly cleaned to remove any leftover materials, grime, and dust. Then, a fine-bristled brush was used to coat the mold's inner surfaces with a release agent. The release agent was allowed to cure and form a non-stick coating by leaving the mold in direct sunlight for about 30 minutes. This coating simplified demolding by reducing the cured laminate's adhesion to the mold surface.

To create a smooth base layer and minimize air entrapment, a uniform layer of the prepared epoxy resin mixture was applied to the mold's surface with a brush. Then, the epoxy layer was covered with bio-filler made of *Aquilaria agallocha* gum, which was followed by an equal volume fraction of hemp and snake grass fibers. Effective reinforcement throughout the composite structure was accomplished by carefully dispersing both fibers and filler particles evenly in order to avoid agglomeration (Table 1).

**Table 1:** Designation of bio-composites

S. no	Fibre content		Total fiber content %	*Aquilloria agallocha* (filler) content%	Epoxy content%	Designation
	Snake grass	Hemp				
1	2.5	2.5	5	15	80	05%SG-HP+E/15%AA
2	5.0	5.0	10	15	75	10%SG-HP+E/15%AA
3	7.5	7.5	15	15	70	15%SG-HP+E/15%AA
4	10.0	10.0	20	15	65	20%SG-HP+E/15%AA
5	12.5	12.5	25	15	60	25%SG-HP+E/15%AA
6	15.0	15.0	30	15	55	30%SG-HP+E/15%AA

To eliminate extra resin, remove imprisoned air bubbles, and distribute the resin evenly, rollers were utilized after arranging the reinforcing materials. This procedure lowered the production of resin-rich areas that may have a detrimental impact on mechanical qualities and improved fiber wetting.

A top cover was placed to seal the mold, and the laminate underwent compression molding. A uniform pressure of 5 bar was applied to compress the composite layers and improve bonding between the matrix, fibers, and bio-filler. During compression, the composite was cured at ambient temperature. Once curing was complete, the pressure was slowly released from 5 bar, allowing the laminate to stabilize before carefully removing it from the mold.

In order to avoid damage or deformation, the cured composite laminate was cautiously removed from the mold. After that, the laminate was either dried in a low-humidity environment or in a temperature-controlled oven in order to eliminate any leftover moisture that might impair its performance and longevity.

The composite panels were 250 mm × 250 mm × 3 mm in size and were later sliced into standard test specimens for mechanical characterization. This methodical manufacturing technique ensured a uniform distribution of filler, effective fiber reinforcement, and consistent composite quality, making it appropriate for extensive experimental evaluation and possible large-scale manufacturing applications.^22–24^

## Mechanism of bonding

Epoxy resins are characterized by their epoxy equivalent weight, which is the resin weight per epoxide group. Bisphenol A Diglycidyl Ether (DGEBA) has a theoretical molecular weight of 340, yielding a theoretical equivalent weight of 170. This value is important for calculating the stoichiometric amine required for optimal curing. TETA (Triethylene Tetramine) is a ligand with two primary and two secondary amine groups, providing six active hydrogens. When primary amines react with epoxy, they form secondary amines, which can then react with additional epoxy molecules, leading to a crosslinked network. Plant fiber composites consist of reinforcing fibers in a polymer matrix, and their performance depends on the matrix and the fiber-matrix bond (Table 2). This bond can involve interdiffusion, electrostatic adhesion, chemical reactions, and mechanical interlocking, all of which are crucial because they allow the matrix to penetrate the fiber's surface. The interfacial characteristics are influenced by hydrogen bonds between cellulose's –OH groups and water.^25–27^

## Composite designation

**Table 2:** Chemical composition of fibres/fillers used in this study^28–32^

S.No	Hemp	Snake grass	*Aquilaria agallocha*
1	Cellulose-70%	Cellulose 56%	Sesquiterpenoids and chromonones 60–70%
2	Hemicellulose-20%	Hemicellulose 34%	Polysaccharide gum 10–20%
3	Lignin-7%	Lignin 6%	Phenolic compounds 5–10%
4	Wax 0.8%	Wax1-2%	Proteins 1–3%
5	Pectin 3%		

## Characterisation

The surface functional group analysis used a Fourier transform infrared spectrometer (FTIR, Bruker tensor 27, Borken, Germany).

Morphological research, chemical composition, and failure mechanisms of biocomposites were studied with Hitachi's SEM and Bruker, Billerica, MA, USA's EDX spectrometer.

## Mechanical testing

Epoxy bio composite samples were tested in accordance with ASTM standards. Tensile testing was performed at 5 mm min^−1^ (ASTM D638); flexural tests were conducted using a three-point bending method at 1.5 mm min^−1^ (ASTM D710); and impact strength was evaluated using IZOD testing (ASTM D256), with results averaged over five samples. Dynamic mechanical analysis (DMA) assessed viscoelastic properties and behavior under various loads and temperatures. Scanning electron microscopy (SEM) provided images for morphological analysis. Water absorption rates were calculated by measuring weight change after immersion in distilled water (ASTM D-570). Biodegradability was tested by burying samples in soil and tracking weight loss over 120 days.

## Result and discussion

### IR spectroscopy

**Fig. 1:**
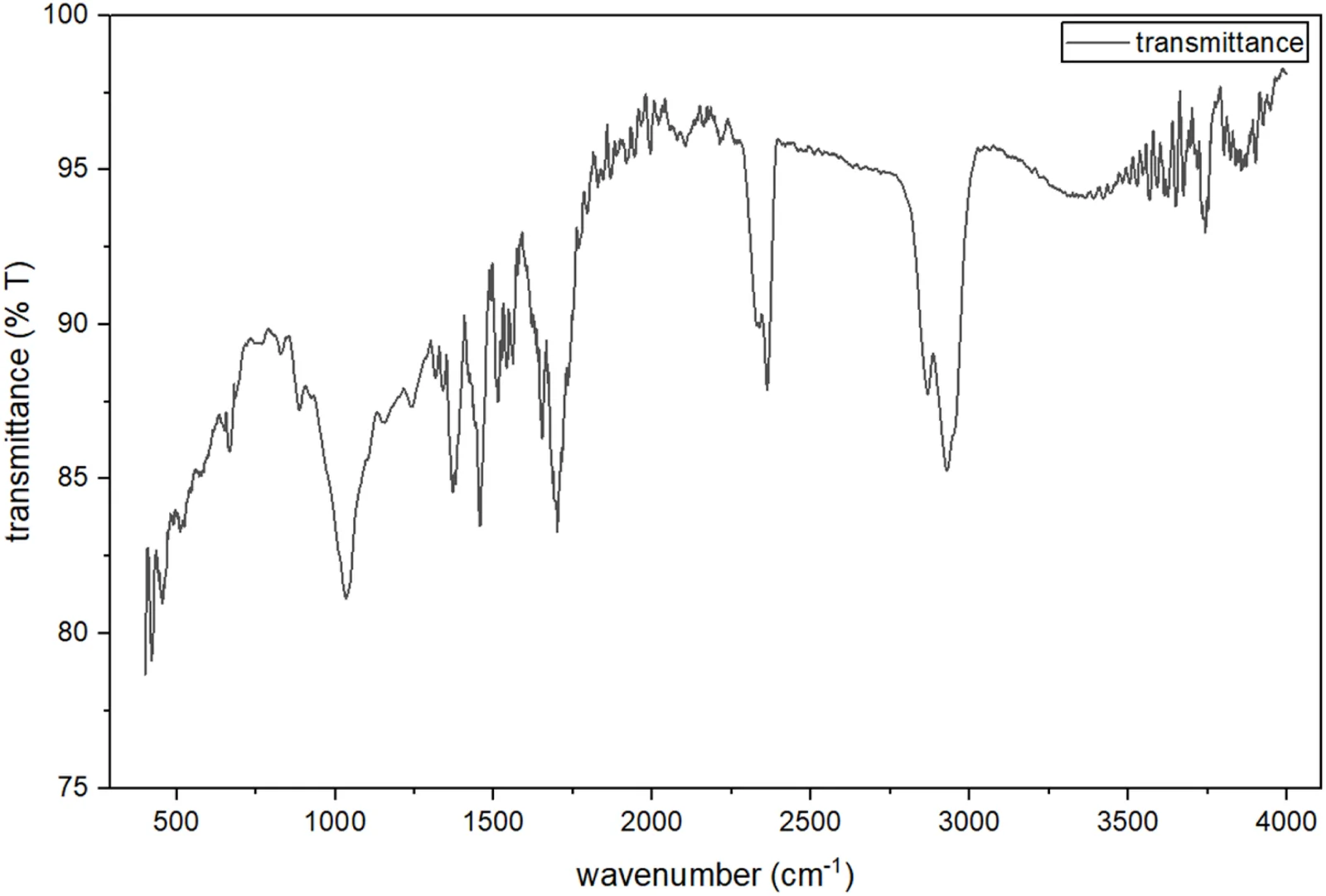
IR Spectrum of 25%SG-HP + E/15%AA.

In Fig. 1 the IR spectrum of the Hemp and Snake Grass and *Aquilaria agallocha* gum-reinforced epoxy resin composite reveal bands associated with epoxy resin, *Aquilaria agallocha* gum, hemp, and snake grass fiber. A broad peak at 3634–3869 cm^−1^ is due to the hydroxyl stretching vibration of H-bonded and free OH groups found in fibrous polysaccharides. The –OH functional group appears in the produced epoxy biocomposite, and its presence is represented by the band at approximately 3634 cm^−1^ in the hybrid biocomposite. The fibers are composed of lignin, hemicellulose, and cellulose. The polymer chain contains several –OH groups, and because this increases contact between the resin and the fibers, it may strengthen their bond to the resin. The targeted application of epoxy polymers to attain suitable fiber wettability serves as the basis for the reinforcement of epoxy *Aquilaria agallocha* composites with snake grass fiber and hemp fiber. The absence of the 914 cm^−1^ peak shows that this improves the interaction between fillers and fibers, which results in interpenetrating polymer systems, stronger interfacial bonding, and increased crosslinking. –CH_2_ – vibrations linked to cellulose and hemicellulose molecules are responsible for the peaks seen at 2928 cm^−1^. The frequencies at 1636, 1668, and 1683 cm^−1^ correspond to chelated flavonoid groups present in *Aquilaria agallocha* gum. The C=O stretching of the glycosidic linkage is indicated by the peak observed at 1036 cm^−1^, which corresponds to the presence of cellulose. The stretching of C–O–C in polysaccharides is indicated by the peak at 1441 cm^−1^.^33^

**Fig. 2:**
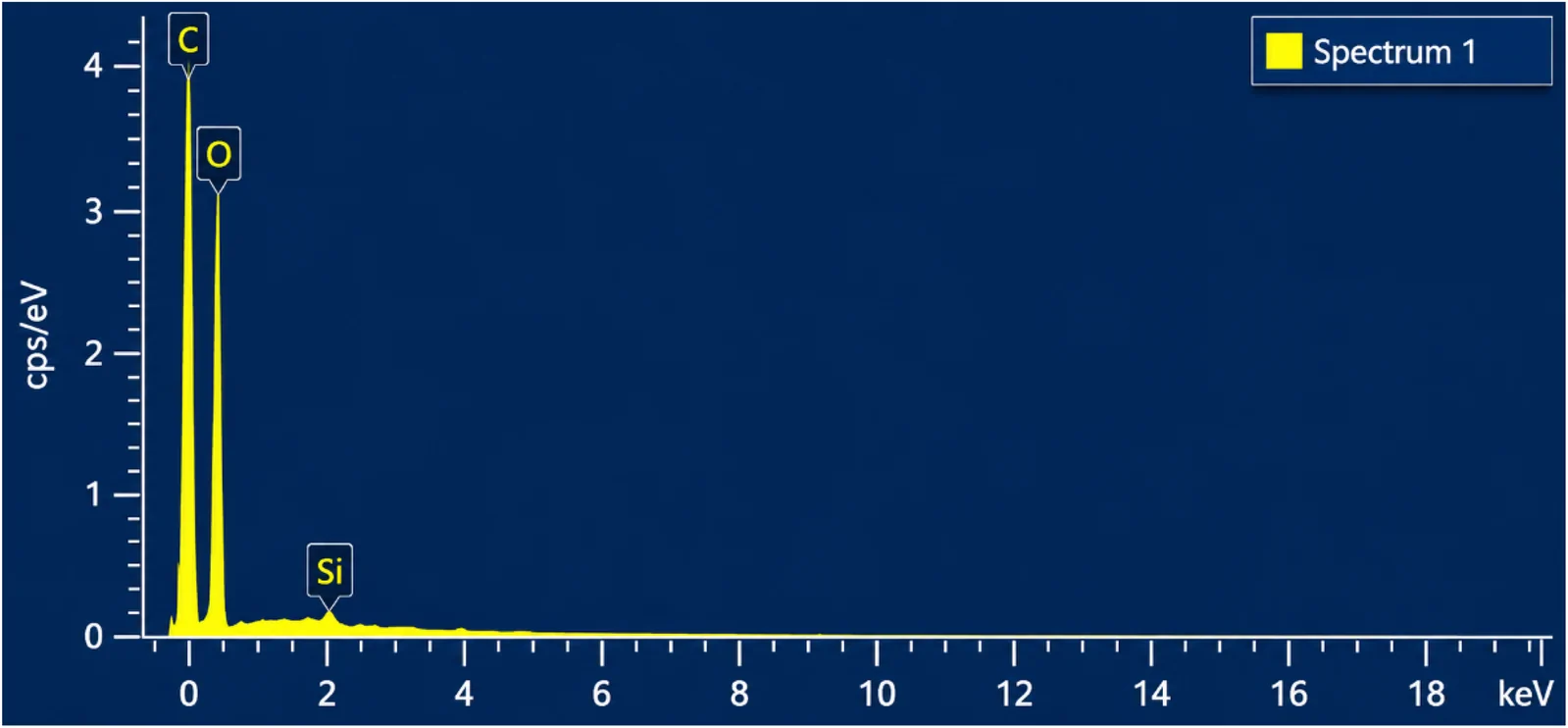
Energy-dispersive X-ray spectrum of 25%SG-HP + E/15%AA.

The EDX spectrum of the hybrid epoxy biocomposite confirms that carbon (C) and oxygen (O) are the dominant elements, consistent with the organic nature of the epoxy matrix, hemp and snake grass fibers, and the *Aquilaria agallocha* biofiller (Fig. 2). The high carbon content arises from the polymer backbone and the lignocellulosic components of the natural fibers, while oxygen is associated with functional groups such as hydroxyl (–OH), ether (C–O–C), and carbonyl (C=O) present in both the epoxy network and the plant-based reinforcements.

The absence of significant metallic or inorganic elemental peaks indicates that no external mineral fillers or metallic reinforcements were incorporated into the composite, confirming its fully organic character. A minor silicon (Si) signal is observed and is attributed to external contamination or to the sample holder/substrate used during SEM analysis rather than to the composite itself.

The C/O ratio reflects the material's organic-rich composition and is consistent with a polymer–fiber hybrid system. The presence of oxygen-containing functional groups supports the possibility of hydrogen bonding and polar interactions at the fiber–matrix interface, which contribute indirectly to improved interfacial adhesion, as evidenced by mechanical and morphological results.

Overall, the EDX results confirm the successful formation of a carbon-based polymer composite reinforced with natural fibers, with no evidence of unintended inorganic fillers or metallic contaminants.^34^

### X-ray diffraction

**Fig. 3:**
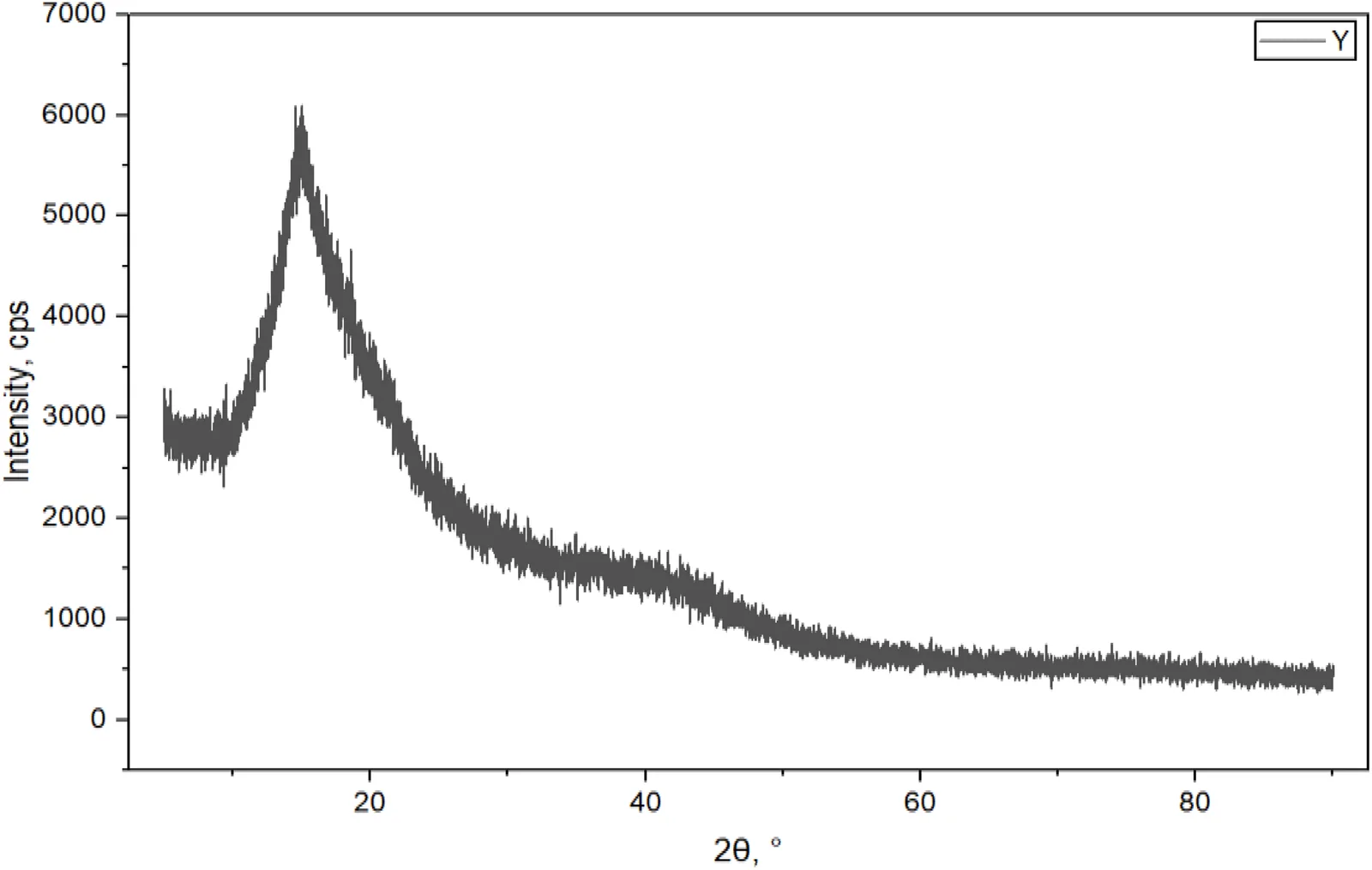
XRD analysis of 25%SG-HP + E/15%AA.

The X-ray diffraction pattern of the 25%SG-HP + E/AA hybrid composite shows a largely amorphous structure marked by a wide diffraction halo around 14.86°. The non-crystalline components of *Aquilaria agallocha* gum and the amorphous epoxy matrix are the main reasons for this wide peak. At greater diffraction angles, faint diffraction peaks are observed, indicating the presence of ordered cellulose domains in the natural fibers. These peaks' comparatively low intensity suggests that the crystalline cellulose structure is spread across a mainly amorphous polymer network. The data indicates that adding natural fibers and gum from *Aquilaria agallocha* does not cause major crystallization in the epoxy matrix. The retained cellulose crystallinity contributes to reinforcement and stiffness, while the predominance of amorphous areas is advantageous for energy absorption and toughness. The DMA results, which show increased stiffness and constrained molecular movement as a result of fiber reinforcement, are in line with the XRD findings (Fig. 3).

### Morphological properties

In order to correlate microstructural features with mechanical performance, scanning electron microscopy (SEM) was used to study the fracture morphology of epoxy composites containing *Aquilaria agallocha* gum biofiller and reinforced with hemp and snake grass fiber.

**Fig. 4:**
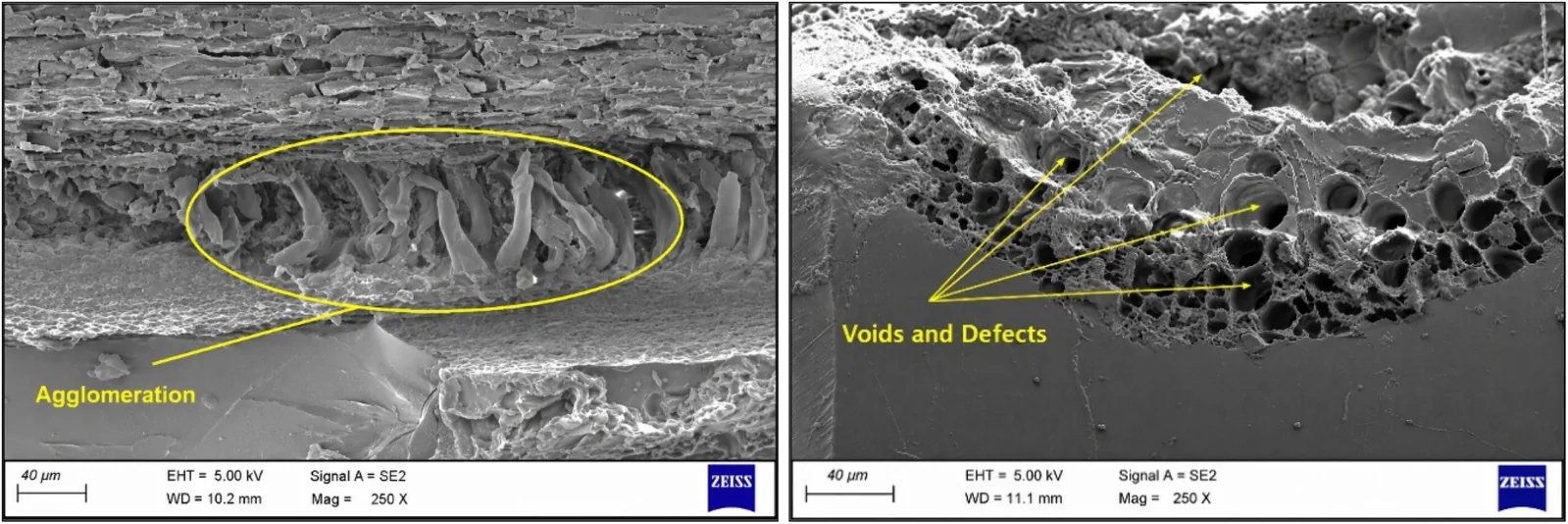
SEM images of 20% SG-HP + E/15%AA.

The 20 vol% SG-HP + E/AA composite shows fairly uniform fiber distribution, good matrix wetting, and little void development. Effective interfacial adhesion and efficient stress transmission between the epoxy matrix and reinforcing fibers are shown by the fact that the majority of fibers are still implanted in the matrix. The highest tensile strength is observed at this composition because there is evidence of matrix deformation and restricted fiber pull-out on the fracture surface. The balanced fiber distribution enables efficient load transmission while reducing locations of stress concentration (Fig. 4).

**Fig. 5:**
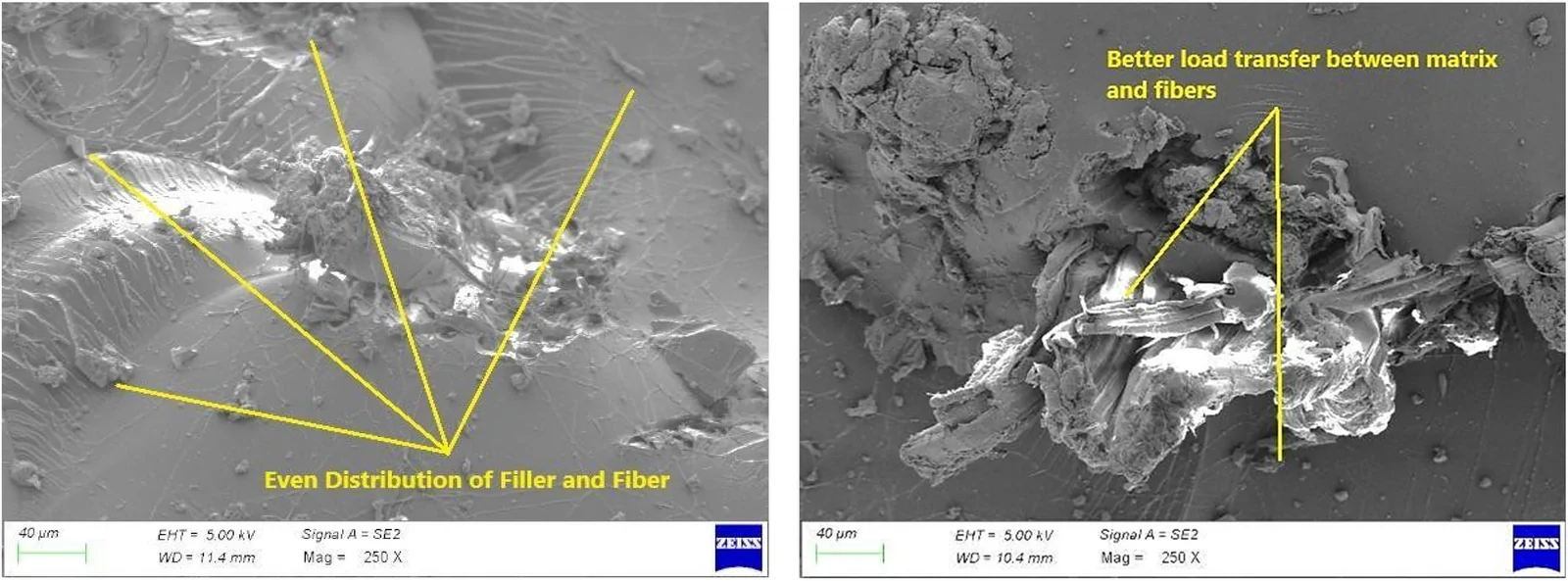
SEM images of 25% SG-HP + E/15%AA.

The 25 vol% SG–HP + E/AA composite displays (Fig. 5) an improved microstructure, with denser and more connected elements, improved fiber packing, and stronger interactions between the fibers and the matrix. Improved energy dissipation mechanisms are indicated by matrix-coated fibers, decreased interfacial gaps, and mixed-mode fracture features. The maximal impact strength seen at this composition may be attributed to fiber bridging, regulated fiber pull-out, and crack deflection, which are important factors in impact resistance.

**Fig. 6:**
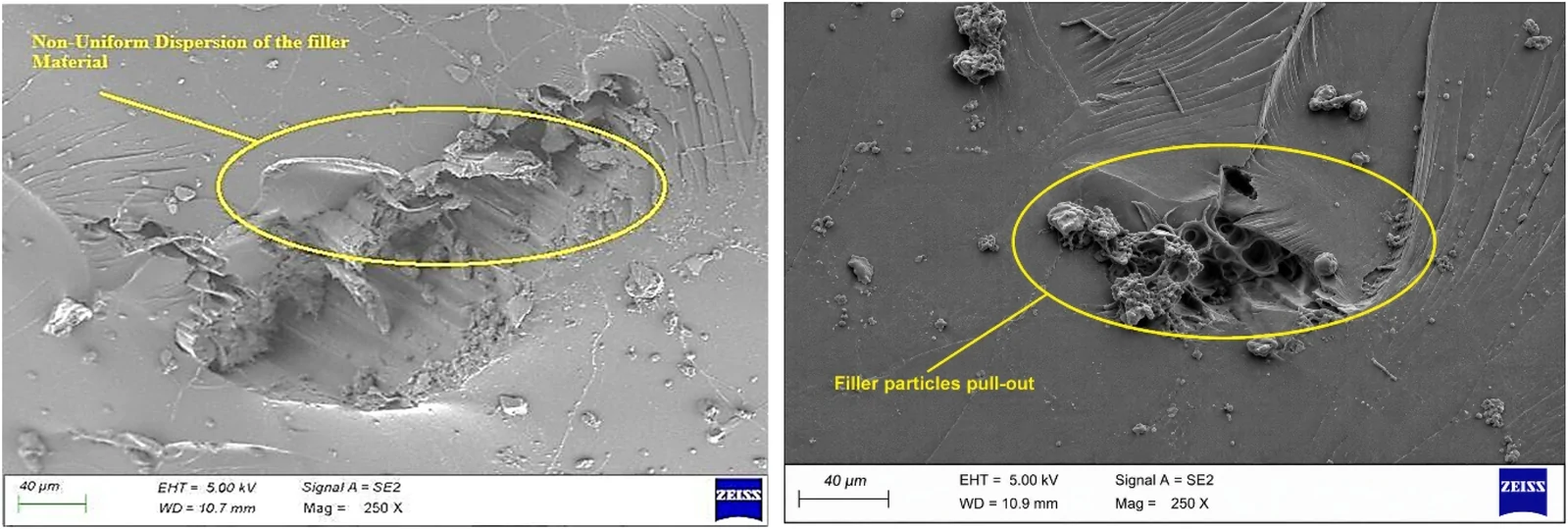
SEM images of 30% SG-HP + E/15%AA.

The microstructure becomes more heterogeneous at 30 vol% fiber loading, exhibiting localized fiber agglomeration, areas devoid of resin, and higher porosity. Even though the increased fiber content helps create a steady load-bearing network that improves flexural stiffness and strength (Fig. 6), too much fiber concentration lowers wetting efficiency and increases interfacial debonding. Under tensile stresses, these flaws promote the start and spread of fractures.

SEM observations indicate that tensile strength is highest at 20 vol% fiber loading because of efficient stress transmission, impact resistance is highest at 25 vol% because of improved energy absorption mechanisms, and flexural performance is enhanced by the bigger fiber network established at 30 vol% fiber loading. The mechanical and dynamic mechanical findings are in close agreement with these microstructural findings.^35^

### Mechanical properties

To develop structure–property relationships as a function of fiber loading, the mechanical performance of epoxy hybrid composites reinforced with hemp and snake grass fibers and *Aquilaria agallocha* biofiller was evaluated in terms of tensile, flexural, and impact properties. The immaculate epoxy performed the worst mechanically because of its fragile, tightly crosslinked structure. Tensile properties improved only marginally with the addition of 15 vol% *Aquilaria agallocha* biofiller, indicating that the particulate phase by itself does not significantly contribute to load-bearing. By contrast, the high cellulose content, stiffness, and effective stress transfer capability of hemp and snake grass fibers led to substantial improvements in all mechanical characteristics when hybridized.

**Fig. 7:**
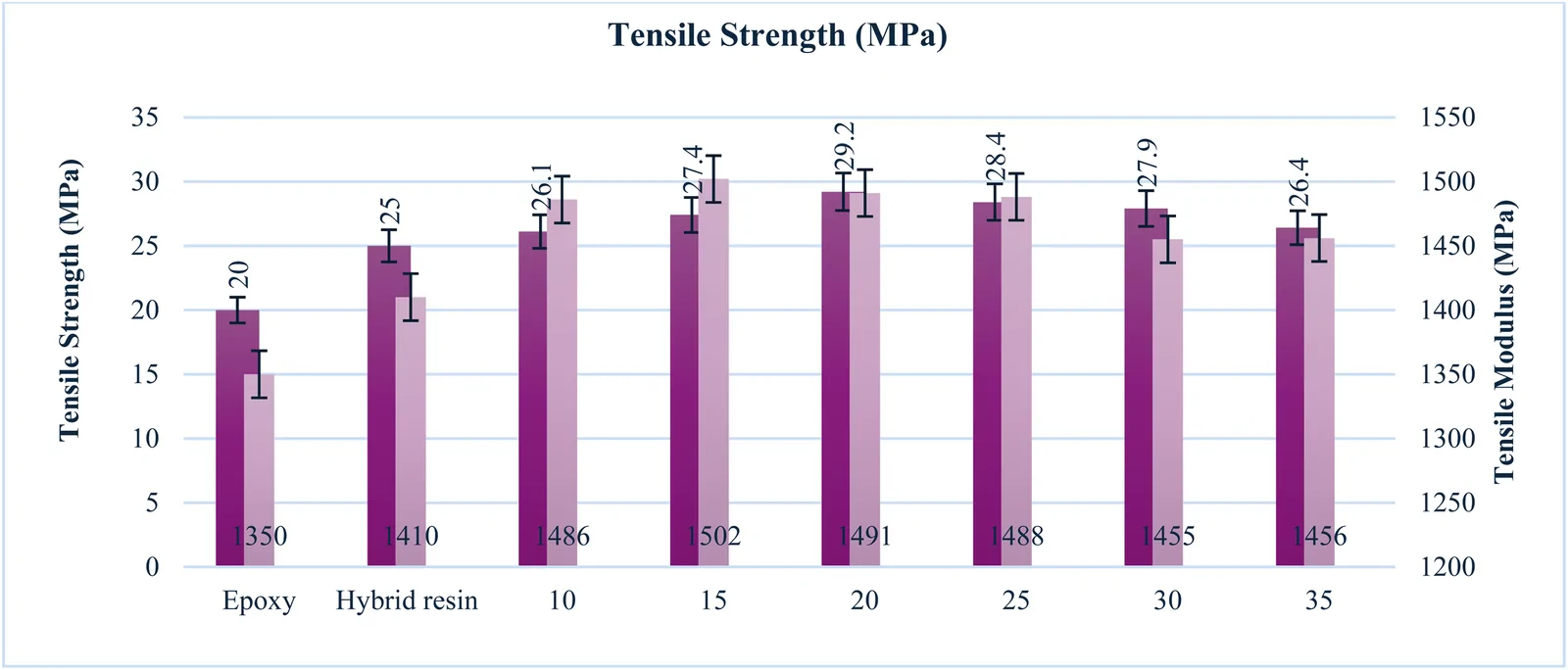
Tensile strength of the composites with varying V/V% of (SG-HP)+E/15%AA.

A fiber content of 20 vol% produced a peak tensile strength of 29.2 MPa, which increased with fiber loading (Fig. 7). This improvement is due to a uniform fiber distribution, strong interfacial bonding, and efficient load transfer between the fiber and matrix. Beyond this optimum, tensile strength decreased because of fiber agglomeration, poor wetting, void formation, and interfacial debonding, which act as stress concentration sites.^36^

**Fig. 8:**
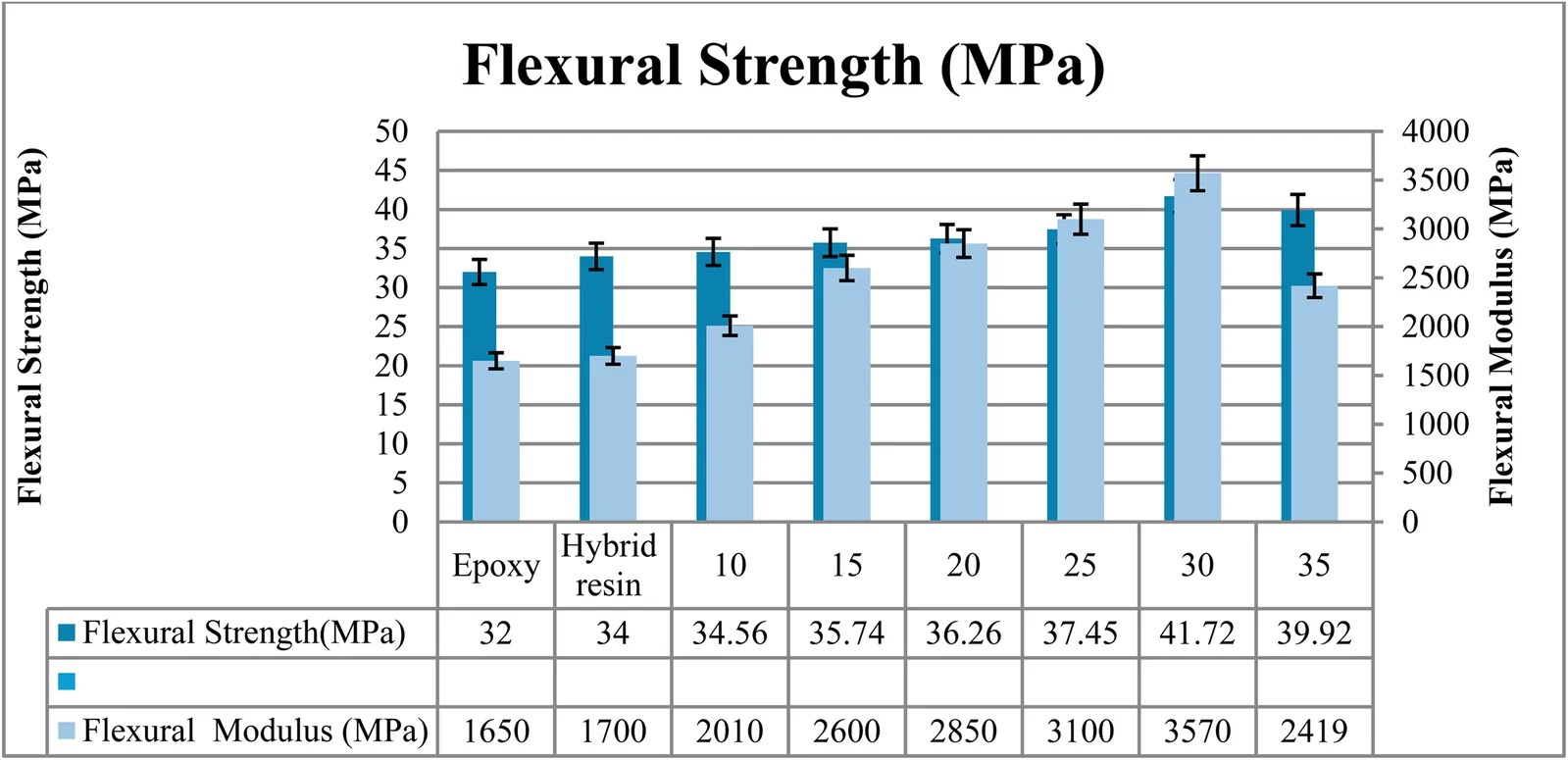
Flexural strength of the composites with varying V/V% of (SG-HP)+E/15%AA.

The flexural strength increased continuously with fiber content, reaching a peak of 41.72 MPa at 30 vol% (Fig. 8). This behavior is attributed to the formation of an efficient fiber network that enhances resistance to bending stresses and improves the outer composite layers' load-bearing capacity. Because aligned fiber regions contribute more to stiffness, flexural performance gains with increased fiber content are greater than those under tensile loading, as shown by the progressive improvement.^37^

At 25 vol% fiber loading, impact strength increased comparably, reaching an optimal value of 2.7 kJ m^−2^. Crack deflection, fiber bridging, and fiber pull-out are energy-absorption mechanisms that contribute to this improvement. At fiber concentrations beyond the ideal, agglomeration and weak interfacial regions occur, impairing the composite's ability to efficiently absorb impact energy (Table 3).

**Table 3:** Comparative account of mechanical properties of fibers and fillers under study

Composite specimen	Tensile strength [MPa]	Tensile modulus [MPa]	Flexural strength [MPa]	Flexural modulas [MPa]	Impact strength [kJ m^−2^]
E+15% AA + 5% (SG + HP)	25.0	1410	34.00	1700	1.5
E+15% AA + 10% (SG + HP)	26.1	1486	34.56	2010	2.0
E+15% AA + 15% (SG + HP)	27.4	1502	35.74	2600	2.5
E+15% AA + 20% (SG + HP)	29.2	1491	36.26	2850	2.6
E+15% AA + 25% (SG + HP)	28.4	1488	37.45	3100	2.7
E+15% AA + 30% (SG + HP)	27.9	1455	41.72	3570	2.4
E+15% AA + 35% (SG + HP)	26.4	1456	39.92	2419	2.2

In general, the results suggest that mechanical properties are governed by a trade-off between the effectiveness of fiber reinforcement and the integrity of the interface. While slightly higher fiber content favors flexural stiffness through network formation, the optimal fiber loading range (20–25 vol%) provides the best balance of tensile and impact resistance. The structural performance of epoxy composites is greatly enhanced by hybridizing snake grass and hemp fibers with biofiller when appropriately optimized, as confirmed by the observed trends.

Because each loading mode has its own dominant failure and deformation processes, the optimal fiber loading for tensile, flexural, and impact characteristics varies. The maximum tensile strength is achieved at 20 vol% fiber loading due to effective fiber–matrix interfacial adhesion and fiber dispersion, enabling efficient axial stress transfer. At higher concentrations, fiber agglomeration and insufficient resin wetting reduce load-bearing efficiency.

**Fig. 9:**
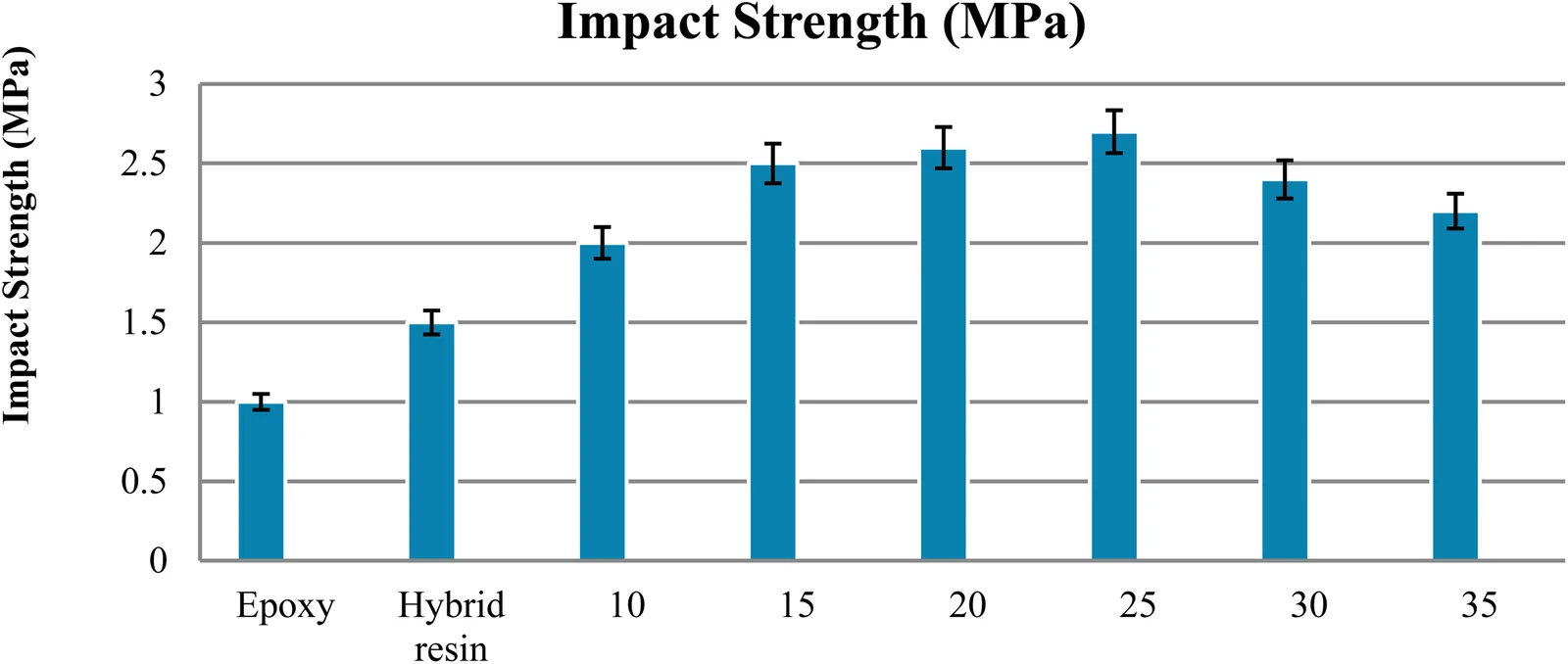
Impact strength of the composites with varying V/V% of (SG-HP)+E/15%AA.

The impact strength peaks at 25 vol% fiber loading because higher fiber concentrations favor energy-absorption mechanisms such as fiber pull-out, crack deflection, and fiber bridging, which increase resistance to abrupt fracture. At this stage, reinforcement and controlled interfacial debonding are balanced, promoting toughness (Fig. 9).

Due to greater stiffness and the formation of a more continuous fiber network within the matrix, the flexural strength peaks at 30 vol% fiber loading. The regions with the highest fiber concentration on the outside of a bend endure the greatest stress, and increased fiber content boosts resistance to deformation. However, exceeding this point leads to void formation and poor wetting, which lowers performance.

Therefore, variations in optimal fiber content result from the interaction between defect generation and reinforcement efficiency, both of which depend on the type of mechanical loading.

Table 4 compares the current hybrid epoxy biocomposite with previously published natural fiber-reinforced epoxy systems that include both plant-based fillers and dual fibers. It is clear that most reported devices employ either single-fiber/filler combinations or dual-fiber reinforcement. In contrast, the current study combines hemp and snake grass fibers with *Aquilaria agallocha* gum to achieve balanced improvements in tensile, flexural, and impact characteristics. This supports the idea that the combined effect of hybrid fiber and filler enhances the material's overall performance.

**Table 4:** Literature comparison

Composites	Tensile strength (MPa)	Flexural strength (MPa)	Impact strength (kJ m^−2^)
Epoxy + Snake Grass + Neem Gum^38^	36.49	65.87	2.68
Epoxy/Kenaf/Snakegrass^39^	59.0	82.1	5.23
Spoxy/Banana/Snakegrass^40^	53.74	130.86	65.42
Epoxy/Jute/Filas(A) + Filas(B)^41^	19.08	63.43	1.899
Epoxy/*Aquilaria*Agallocha/Snakegrass/Hemp	29.2	41.72	2.70
Epoxy/Banana/Sisal/CashewNut Filler^42^	43	92	5.61
Epoxy/Bananastem/Jute/Tamarind Shell Powder^43^	40.37	86.02	3.56
Epoxy/Palmyrapalm/Coconut Sheath/Tamarind Shell Powder^44^	42.22	94.45	10.2J
Epoxy/Banana Fibre/Ricehusk/Egg Shell Powder^45^	17.42	25.06	28 610
Epoxy/Dammar Gum/Arecanut Husk/Banana Fiber^46^	24.23	47.89	—
Epoxy/Ramie Fiber/Rice husk/Syzygium fillers and Epoxy/RamieFiber/Cinnamon inner filler/Syzygium fillers [filler mixtures]^47^	26.6	45.66	—
	28.10	37.22	—
Epoxy/Sisal/Bagasse/Coir^48^	53.25	3150	0.065
Epoxy/Borassus flabellifer fruit fiber; snake grass fiber; gum Arabic^49^	72	89	22
Epoxy/Hemp/Coir/Nano egg shell^50^	44	87	—

The Epoxy/*Aquilaria*/Hemp/Snake Grass composite has tensile strength between that of certain natural fiber epoxy hybrids. It outperforms jute and some banana systems but is weaker than borassus and kenaf composites. Its flexural strength is moderately high, showing good load-bearing ability, but its impact strength is lower than modern hybrids with nano-fillers. Strength characteristics improve with fiber hybridization, but optimization is needed for better impact resistance and toughness (Table 5).

**Table 5:** DMA parameters of the composites with varying V/V% of (SG + HP)+ E/15%AA (estimated from graphs)

Specimen name	Onset of *E*′ (°C)	Peak of *E*″ (°C)	Peak of tan *δ* (°C)	Peak height of tan *δ*
Epoxy	∼50	∼60	∼58–60	∼0.85
5 vol% SG + HP	∼52	∼62	∼62–65	∼0.80
10 vol% SG + HP	∼55	∼65	∼68–70	∼0.75
15 vol% SG + HP	∼58	∼68	∼72–74	∼0.70
20 vol% SG + HP	∼60	∼72	∼75–77	∼0.65
25 vol% SG + HP	∼62	∼75	∼78–80	∼0.60
30 vol% SG + HP	∼60	∼73	∼76–78	∼0.62
35 vol% SG + HP	∼58	∼70	∼72–75	∼0.65

### Dynamic mechanical analysis (DMA)

#### Cole–Cole plot

The interfacial qualities of composites, particularly between the epoxy filler matrix and the fibers, have a significant impact on their viscoelastic properties. The geometry and surface roughness of the fibers are important variables in determining the viscoelastic and interfacial properties of composites made with natural fibers. Natural fiber structures are typically complex, consisting of numerous layers, including cellulose, hemicellulose, lignin, and waxes.

DMA was used to examine the viscoelastic behavior and interfacial properties of the developed composites by measuring Cole–Cole plots, damping factor (tan *δ*), loss modulus (*E*″), and storage modulus (*E*′).

**Fig. 10:**
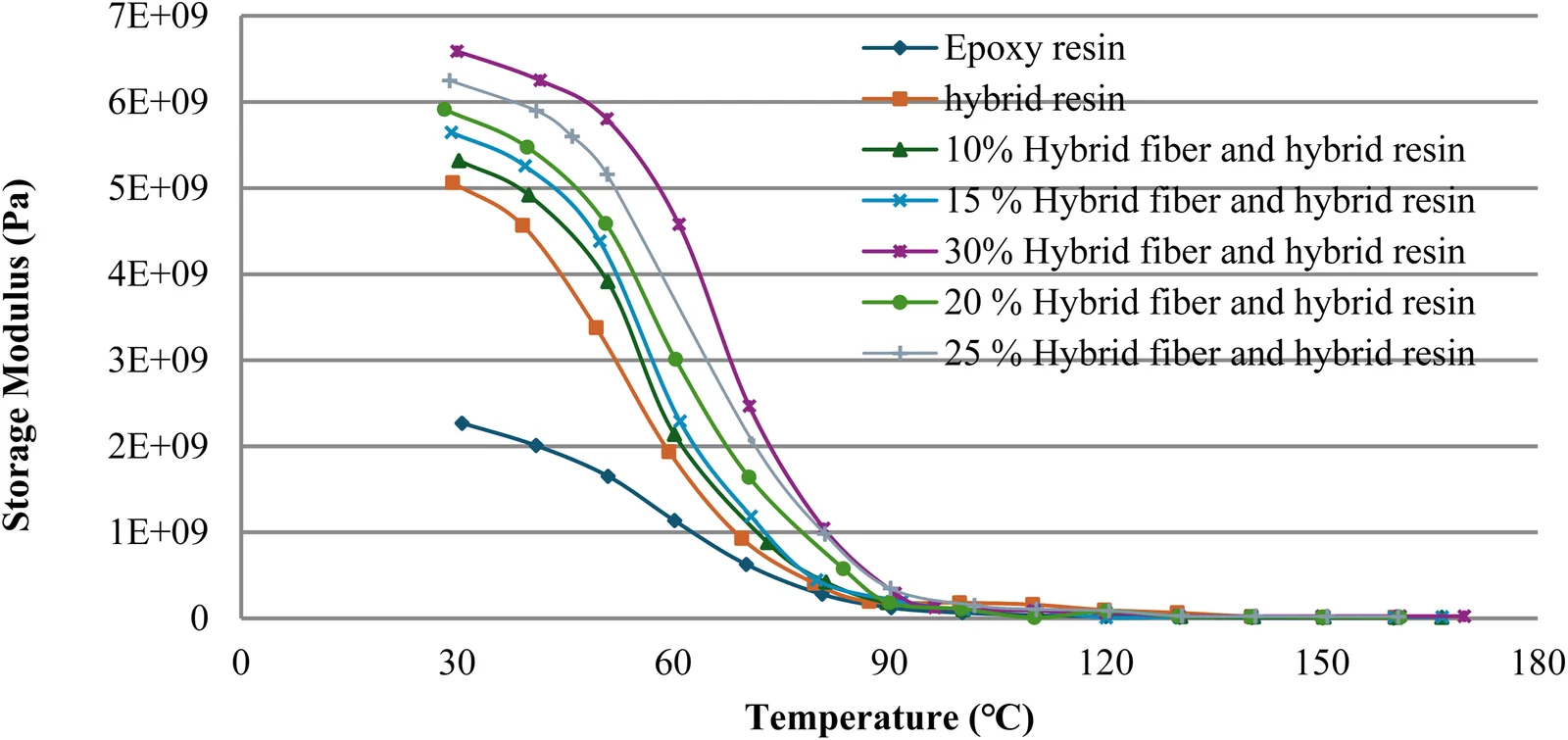
Storage modulus of the biocomposites with varying V/V% of (SG + HP) + E/15%AA. at 10 Hz frequency.

The elastic response and stiffness of the substance are shown by the storage modulus (*E*′). Over the entire temperature range, the storage modulus was lowest in neat epoxy. The addition of *Aquilaria agallocha* gum, along with hemp and snake grass fibers, increased *E*′ significantly because of the higher stiffness of the cellulose-rich fibers and the improved stress transfer at the fiber-matrix interface. The storage modulus increased progressively as the fiber loading increased, reaching a peak value of about 6.5 × 10^9^ Pa at 30 vol% fiber loading (Fig. 10). The reinforced efficiency is improved and the polymer chain motility is limited by this increase. Fiber agglomeration and interfacial faults may be responsible for the slight decrease in modulus outside the ideal area.

The loss modulus (*E*″) indicates the amount of energy lost during cyclic deformation. All composite systems displayed a distinct *E*″ peak that corresponded to the glass transition area. Increased interfacial friction and energy dissipation resulting from fiber-matrix interactions and localized molecular rearrangements are shown by the rise in loss modulus with fiber loading.

**Fig. 11:**
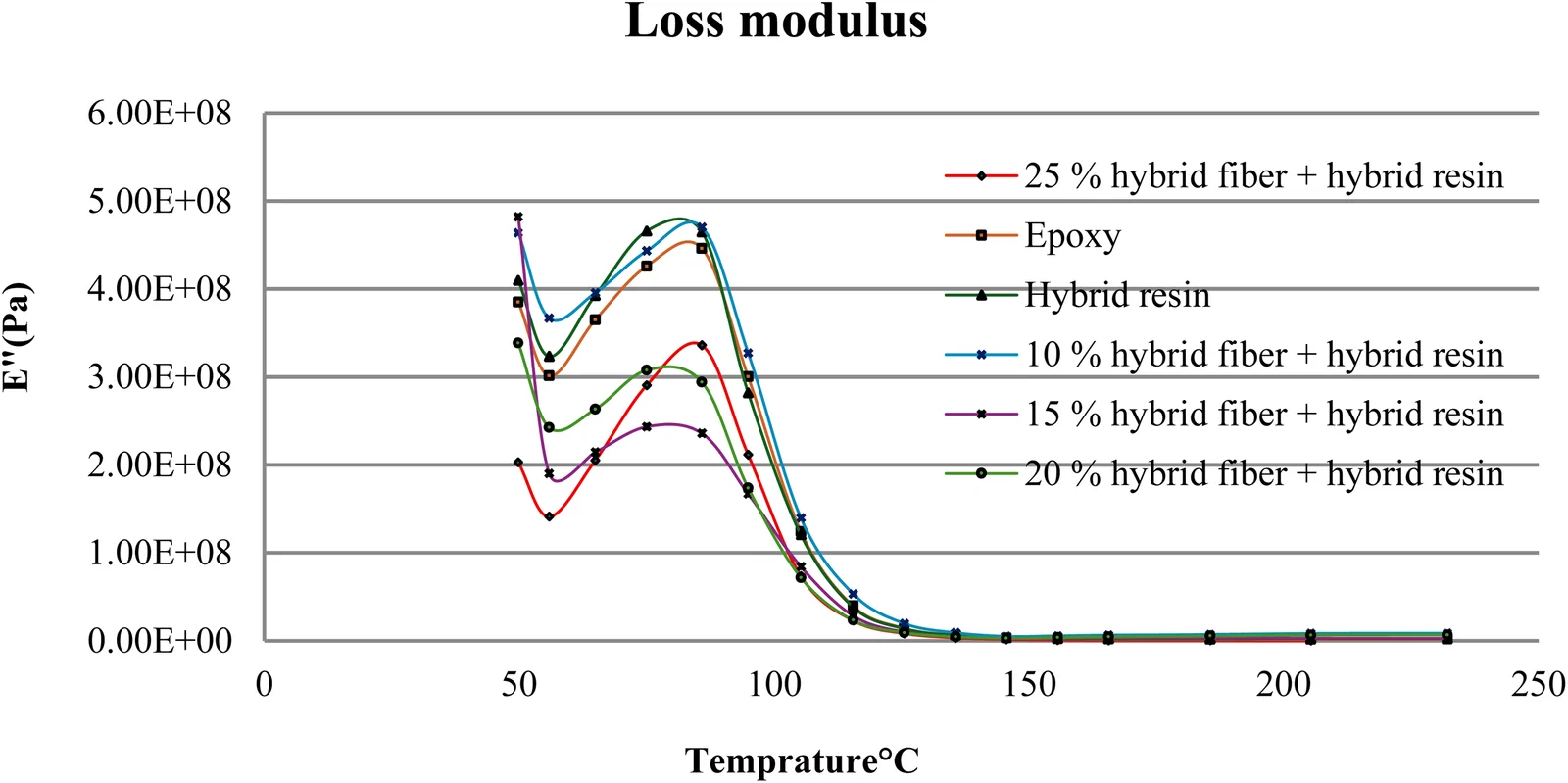
Loss modulus of the biocomposites with varying V/V% of (SG + HP)+ E/15%AA at 10 Hz frequency.

Only from the tan peak temperature was the glass transition temperature (*T*_g_) determined. The *T*_g_ of pure epoxy was between 58 and 60 °C, while the *T*_g_ values of the hybrid composites increased with increasing fiber content. The 25 vol% SG-HP + E/AA composite had a maximum *T*_g_ of about 78–80 °C. Due to enhanced interfacial adhesion and reinforcement effects, this movement toward higher temperatures verifies limited segmental movement of the epoxy chains. Localized heterogeneity and incomplete wetting may cause a small decrease in *T*_g_ at higher fiber contents (Fig. 11).

**Fig. 12:**
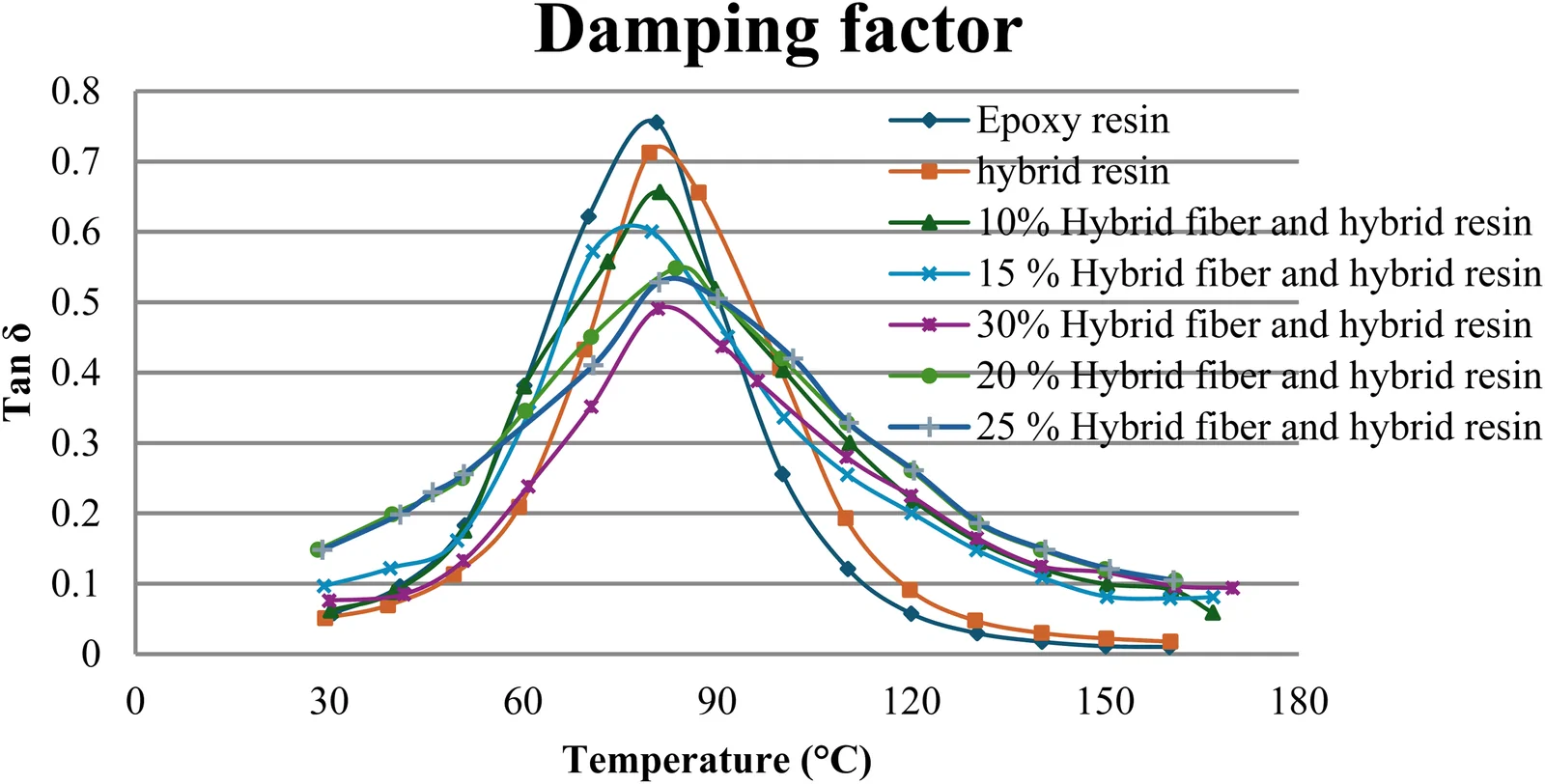
Damping factor of the composites with varying V/V% of (SG + HP)+ E/15%AA.

Reduced molecular mobility and improved elastic behavior were shown by a reduction in the damping factor (tan *δ*) as the fiber content increased. Lower tan *δ* readings point to more effective stress transfer inside the composite structure and greater fiber-matrix interactions (Fig. 12).

**Fig. 13:**
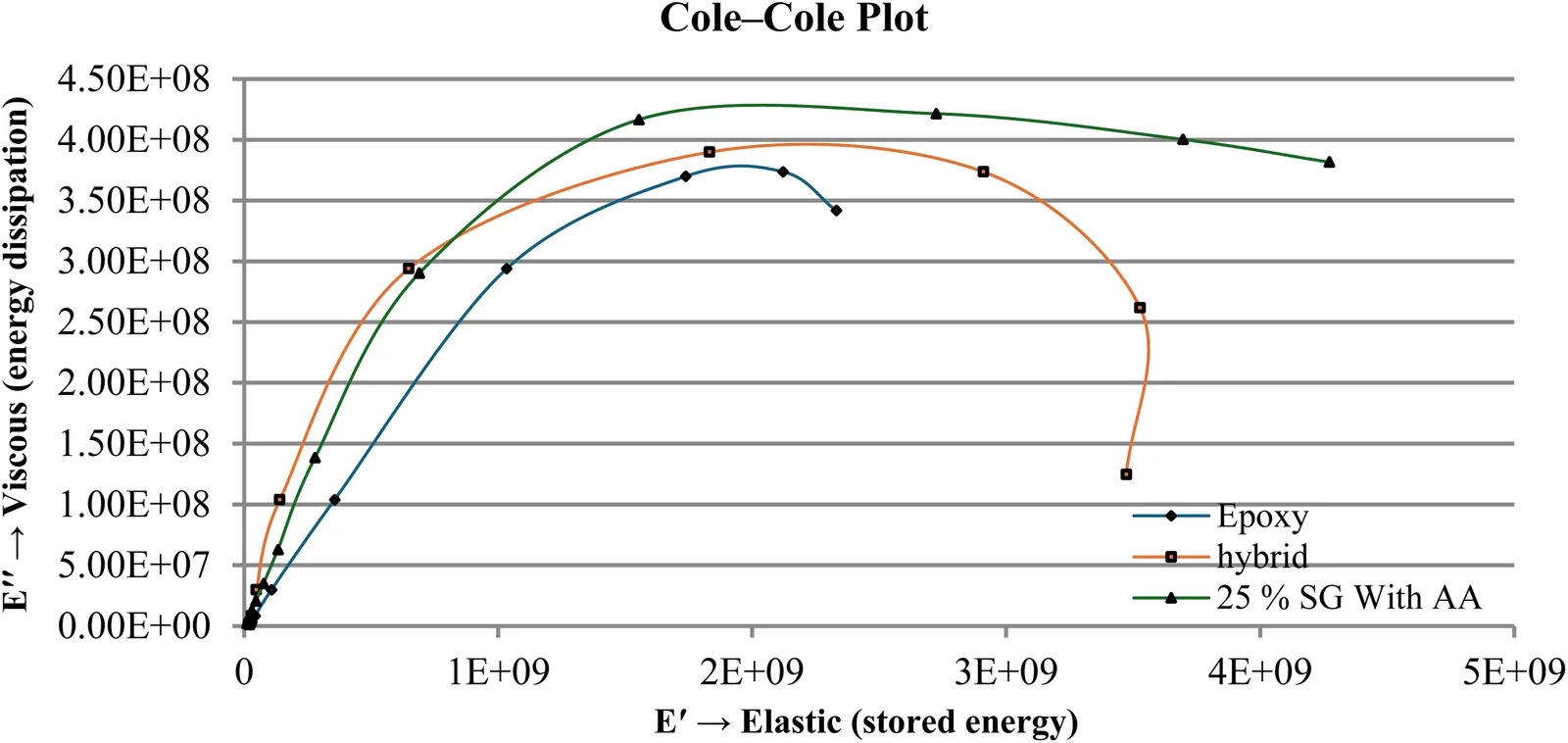
Cole–Cole plot with Hemp and SnakeGrass + E/15%AA hybrid matrix polymer composite specimens.

The composite system's homogeneity was evaluated using Cole–Cole plots (Fig. 13). The Cole–Cole plot in a perfectly homogeneous viscoelastic material shows an ideal semicircular form that corresponds to a single relaxation process. Structural heterogeneity and numerous relaxation processes are indicators of semicircularity departures. Fairly homogeneous relaxation behavior was shown by the virtually semicircular shape of the neat epoxy. The curves became gradually distorted and flattened as *Aquilaria agallocha* filler and hybrid fibers were incorporated. The existence of various relaxation systems that are related to fiber-matrix interactions, localized interfacial zones, and fiber–fiber contacts is confirmed by this activity. The greatest departures were found at higher fiber loadings, which suggests a rise in structural heterogeneity.

In general, the results of DMA show that the interfacial interactions, thermal stability, and stiffness are greatly improved by hybrid reinforcement. At 20 vol% fiber loading, tensile performance was maximized; at 25 vol%, impact resistance was maximized; and at 30 vol%, flexural performance and maximum storage modulus were maximized, demonstrating property-dependent optimization of the composite system.

## Water absorption studies

Due to the inherently hydrophilic nature of natural fibers, water absorption behavior is a critical parameter governing the durability and service performance of epoxy-based biocomposites. Neat epoxy, owing to its hydrophobic nature and dense crosslinked structure, exhibits minimal water uptake. In contrast, incorporating *Aquilaria agallocha* (AA) biofiller and hybrid hemp (HP) and snake grass (SG) fibers introduces hydrophilic functional groups (–OH groups in cellulose and hemicellulose), which increase moisture affinity.

The water absorption behavior of the composites increases progressively with fiber loading due to greater availability of polar sites and larger interfacial regions. At lower fiber contents (5–10 vol%), moisture uptake remains relatively low because the epoxy matrix sufficiently encapsulates the fibers, limiting diffusion pathways. As fiber loading increases to 15–20 vol%, water absorption rises gradually due to a larger fiber–matrix interfacial area and the formation of microvoids, which serve as diffusion pathways for water molecules.

At intermediate loadings (20–25 vol%), a partially improved packing structure and better fiber dispersion lead to more controlled moisture uptake, indicating a balance between hydrophilicity and interfacial integrity. However, at higher fiber contents (30 vol% and above), water absorption increases significantly due to fiber agglomeration, poor matrix wetting, and increased void formation, which facilitate capillary transport and moisture penetration.

**Fig. 14:**
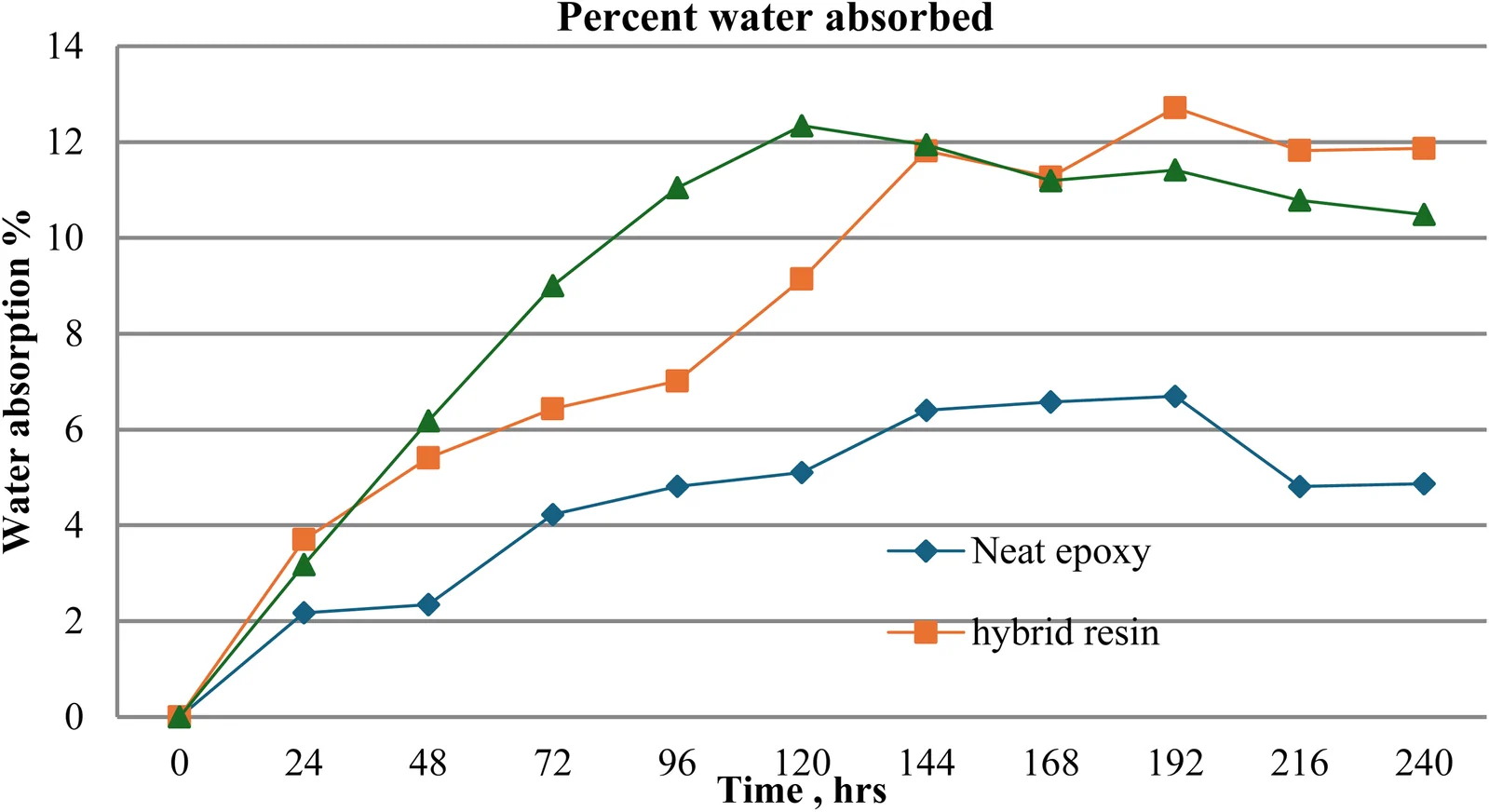
Water absorption studies.

The time-dependent absorption profile shows an increase in water uptake from 2.17% (24 h) to 11.9% (144 h) for the hybrid systems, followed by a tendency toward equilibrium. In some cases, a slight reduction after saturation is observed due to leaching of soluble components and partial structural relaxation, while prolonged exposure may again increase absorption due to swelling-induced microcracking (Fig. 14).

The observed water absorption behavior is strongly correlated with SEM microstructural features. At lower and intermediate fiber loadings, SEM analysis reveals relatively uniform fiber dispersion, improved resin wetting, and fewer voids, all of which restrict water diffusion. In contrast, at higher fiber loadings (25–30 vol%), SEM images show increased fiber pull-out, interfacial debonding, and microvoid formation. These defects act as continuous capillary pathways, significantly enhancing moisture ingress. Thus, both chemical hydrophilicity and morphological defects collectively govern the water absorption behavior of the composites. Overall, the study confirms that water uptake in hybrid composites is governed by fiber content, interfacial bond quality, and microstructural porosity, with optimal resistance observed at intermediate fiber loadings.

The findings correlate with the findings Ayyappa Atmakuri *et al.* who reported a similar trend in hemp/flax fiber hybrid composites. But after 168 h E/AA shows an increase in water absorption because swellings cause cracks, internal stress and other structural changes which facilitate the absorption of water molecules Rahman, *et al.*.^29,35,51^

### Bio degradation test

The breakdown of epoxy/natural fiber bio composites, along with the loss of mechanical properties, is a sign of deterioration caused by the disintegration of cellulose, hemicellulose, and lignin in the reinforcement fibers. These fiber components are broken down by the combined effects of atmospheric humidity, temperature, pressure, ultraviolet radiation, and the activity of microorganisms. Although the biodegradability of the bio composites is determined by the fiber type, it is evident that natural fibers in an epoxy matrix improve their biodegradability. Compared to fibers with lower hemicellulose concentrations, those with higher hemicellulose content are more biodegradable.

**Fig. 15:**
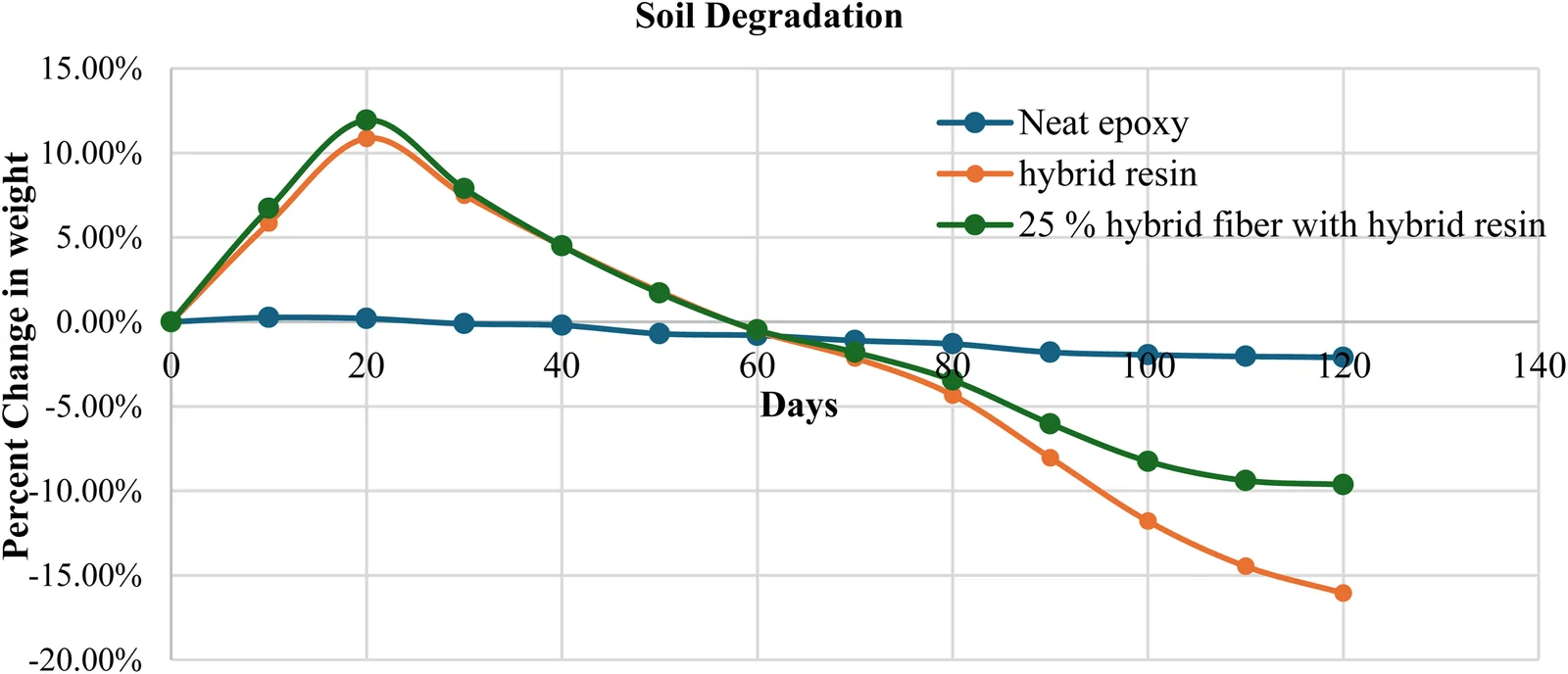
Change in weight of the soil buried gum composites.

Neat epoxy is non-biodegradable and hydrophobic. During the first 20 days, E/AA shows a weight increase of about 11%, and the hybrid fiber epoxy composite shows 12.5%. The weight increase in the composite systems is attributed to moisture and the presence of microbes in the soil. Fibers show a greater increase in weight due to the presence of hemicellulose. After 20 days, the weight of the composite approaches zero. After another 120 days, the E/AA composite shows a decrease in weight of about 16.05%, and the hybrid fiber composite shows 9.63% (Fig. 15). E/AA shows higher mass loss during degradation because of the presence of soluble components, including phenolic components, and the amorphous nature of the filler. Fibers degrade slowly because they contain crystalline cellulose. Owing to their rigid nature, the presence of OH groups, and hydrogen bonding between the fibers and the epoxy matrix, resulting in good interfacial adhesion, fibers show less mass loss during degradation (Sanjay *et al.*, 2021). From the SEM images, it is clear that for 25% fiber loading, interfacial adhesion is higher, hence showing less mass loss during degradation^52,53^

## Conclusions

A sustainable epoxy-based hybrid biocomposite was successfully developed using *Aquilaria agallocha* gum as a biofiller and hybrid reinforcement of hemp and snake grass fibers. The study demonstrated that fiber content plays a critical role in governing the overall performance of the composite system.

Optimum mechanical performance was achieved in the 20–25 vol% hybrid fiber range, where tensile strength (29.2 MPa), impact strength (2.7 kJ m^−2^), and storage modulus (∼6.5 × 10^9^ Pa) were maximized due to efficient fiber dispersion, strong interfacial bonding, and effective stress transfer. Flexural strength reached a maximum of 41.72 MPa at 30 vol% fiber loading, attributed to the formation of a more continuous load-bearing fiber network. Dynamic mechanical analysis confirmed an increase in glass transition temperature to approximately 80 °C, indicating reduced polymer chain mobility and improved thermal stability.

Water absorption and biodegradation studies highlighted the influence of hydrophilic cellulose-rich fibers, showing controlled moisture uptake and measurable mass loss under soil burial conditions, confirming partial biodegradability while maintaining structural integrity. However, fiber loading beyond the optimum led to agglomeration, void formation, and interfacial debonding, which reduced performance.

Overall, the synergistic combination of hemp and snake grass fibers with *Aquilaria agallocha* gum significantly enhances the mechanical and functional performance of epoxy composites. The developed material offers a promising route toward lightweight, sustainable, and environmentally responsible structural applications.

## Conflicts of interest

There are no conflicts to declare.

## Data Availability

All data supporting the findings of this study are included within the article. Additional experimental data related to bio composite characterization and bio composite performance data are available from the corresponding author upon reasonable request.
